# Changes in Workplace Productivity and Estimated Cost Savings During Internet-Based Cognitive Behavioral Therapy in the Irish National Health Service: Naturalistic, Repeated-Measures, Retrospective Survey Study

**DOI:** 10.2196/80689

**Published:** 2026-04-07

**Authors:** Chi Tak Lee, Siobhán Harty, Angel Enrique, Alba Jiménez-Díaz, Garrett Hisler, Daniel Duffy, Derek Richards

**Affiliations:** 1 School of Psychology, Trinity College Dublin, Dublin, Ireland; 2 Trinity College Institute of Neuroscience, Trinity College Dublin, Dublin, Ireland; 3 Global Brain Health Institute, Trinity College Dublin, Dublin, Ireland; 4 Amwell Science, Amwell, 1 Stephen Street Upper, Dublin, D08 DR9P, Ireland, 353 1 554 9771; 5 Department of Personality, Evaluation and Psychological Treatment, Faculty of Psychology, University of Valencia, Valencia, Spain

**Keywords:** digital mental health, internet-based cognitive behavioral therapy, iCBT, workplace productivity, depression, anxiety, cost effectiveness, health services

## Abstract

**Background:**

Depression and anxiety can significantly impact workplace productivity, for instance, by increasing absenteeism and presenteeism. This loss of productivity leads to diminished workplace economic outcomes. Internet-based cognitive behavioral therapy (iCBT) has emerged as a cost-effective intervention within workplace settings that improves workplace productivity loss due to depression and anxiety, but more generalizable evidence beyond the workplace, such as in a national health service setting, is lacking.

**Objective:**

This naturalistic, repeated-measures, retrospective study investigated the impact of iCBT on work productivity metrics using nationally representative data from patients enrolled in the Irish national health service (ie, the Health Service Executive).

**Methods:**

We analyzed repeated measures retrospective data from 7125 employed patients enrolled in iCBT at the Health Service Executive between March 2023 and May 2024. The Work Productivity and Activity Impairment questionnaire was used to measure absenteeism, presenteeism, overall productivity loss, and activity impairment. Secondary outcomes included depression (Patient Health Questionnaire-9) and anxiety (Generalized Anxiety Disorder-7). Patients were primarily 25 to 64 years old (n=5578, 78%), female (n=4956, 70%), and met clinical scoring criteria on the Patient Health Questionnaire-9 or Generalized Anxiety Disorder-7 (n=4774, 67%). Missing data were handled using multiple imputation. We used mixed-effects models to assess pre-post treatment changes in outcomes and then utilized Irish national salary estimates from 2022 to derive cost savings (in 2022 € values; €1=approximately US $1.05) based on productivity improvement during use of the iCBT program.

**Results:**

From baseline to follow-up, absenteeism reduced by 6.85% (*P*<.001, 95% CI 5.79%‐7.91%, Cohen *d*=0.21), presenteeism reduced by 5.84% (*P*<.001, 95% CI 4.59%‐7.09%, Cohen *d*=0.18), productivity loss reduced by 9.48% (*P*<.001, 95% CI 8.30%‐10.66%, Cohen *d*=0.27), and activity impairment reduced by 8.34% (*P*<.001, 95% CI 7.07%‐9.61%, Cohen *d*=0.30). Depression symptom scores reduced by 2.70 points (*P*<.001, 95% CI 2.50‐2.90, Cohen *d*=0.51) and anxiety symptom scores reduced by 2.71 points (*P*<.001, 95% CI 2.51‐2.91, Cohen *d*=0.52) scores posttreatment. Larger reductions in depression and anxiety symptoms were linked to greater improvements in workplace functioning outcomes (*r*=0.06-0.34, all *P*<.001). Patients with higher baseline clinical severity experienced approximately 6% greater improvements in workplace productivity than subclinical patients (*P*<.001). These improvements in work productivity from baseline to follow-up corresponded to more than €4000 annual savings per patient treated, which equated to an estimated annual savings of €29 million in the sample (in 2022 € values). The average currency exchange rate in 2022 would be €1 to approximately US $1.05.

**Conclusions:**

Building on work that primarily focused on work productivity improvements from iCBT in occupational contexts, this study provides evidence that iCBT for the general adult population in a national routine health care setting is associated with improvements in both Common mental disorders (CMD) and workplace productivity losses associated with CMD. These productivity improvements were further associated with sizable cost savings. These findings suggest that integrating iCBT into national mental health infrastructure can be a feasible, scalable solution, which could generate clinical and economic benefits at a population level.

## Introduction

Common mental disorders (CMDs) like depression and anxiety impact millions of people worldwide [1], resulting in negative social, functional, and health outcomes for individuals [2], as well as significant economic costs. Indeed, the global economy suffers an annual loss of US $1.15 trillion due to CMD, with one-third of this loss attributed to lost employment and productivity [3]. Compared to healthy individuals, 4.7 billion additional workdays are lost, amounting to a total cost of US $592 billion per year [3]. These significant implications warrant cost-effective interventions for CMD, with cognitive behavioral therapy (CBT) showing promise as an affordable, accessible solution when delivered online. Internet-based cognitive behavioral therapy (iCBT) has consistently demonstrated effectiveness in treating CMD across various settings [4-7], with cost-effectiveness [8] superior to other treatments or no treatment [9,10]. One key area to determine the cost-effectiveness of iCBT is through the indirect costs of CMD, such as the costs of decreased workplace productivity due to CMD [11]. Interestingly, recent systematic reviews on economic evaluations of iCBT for CMD suggest iCBT economic evaluations typically focus on direct costs (eg, costs of treatment, prescriptions, medical care), and indirect costs are frequently lacking or understudied [8,12].

CMD can accrue indirect costs through the impairment of workplace productivity, such as by increasing absenteeism (increased work absences), presenteeism (reduced effectiveness at work), higher rates of disability leaves, and early retirement [13-15]. These effects are observed at both clinical and subclinical levels of CMD [16,17]. A common method of measuring workplace productivity loss due to health conditions is through the Work Productivity and Activity Impairment (WPAI) questionnaire [18-20]. This questionnaire allows for the assessment of absenteeism, presenteeism, and overall productivity loss (the combination of absenteeism and presenteeism), as well as general activity impairment (the degree to which a health problem impairs daily activities outside of work), though the activity impairment index is less relevant for examining economic consequences. Focusing on the WPAI, a recent systematic review found that depression was associated with approximately 26 work absences per year and a 37% to 52% reduction in effectiveness at work [21].

Research conducted in occupational contexts suggests that iCBT can also improve absenteeism, presenteeism, and overall productivity [22-28], which, in turn, generates substantial financial returns for employers [22-24,29]. However, access to effective interventions through an employer is not always guaranteed. Despite growing awareness and actions addressing employee well-being, adoption and utilization rates of workplace mental health programs are low and dependent on company structure and culture [30,31]. Alternatively, iCBT administered to the general population through a public health service could provide a different approach to improving work productivity losses due to CMD. However, whether the positive impact of iCBT on work-related outcomes can generalize beyond workplace settings remains unclear.

The integration of iCBT into public health services can greatly increase the reach and cost-effectiveness of mental health care nationwide [25]. This expanded reach should potentially amplify any associated benefits from improved workplace productivity. In Ireland, the Health Service Executive (HSE) provides comprehensive, publicly funded mental health services for treating CMD. The HSE incorporated iCBT from *SilverCloud Health* by Amwell as part of their offering in 2021, making it one of the first nationally implemented iCBT services. Previous work on SilverCloud has shown that it is effective in treating CMD within the HSE and has yielded acceptable service-level metrics and high levels of user satisfaction [32]. Outside of the HSE, previous work has supported SilverCloud as being cost-effective within the United Kingdom’s Improving Access to Talking Therapies, though this prior study was not able to incorporate or specifically examine costs associated with workplace productivity loss [33].

Building on this, we investigated the impact of providing iCBT in a national health service on workplace functioning, including absenteeism, presenteeism, productivity loss, and activity impairment. To do so, we analyzed mental health and work productivity routine care data collected from patients enrolled in SilverCloud at the HSE. We hypothesized that both mental health symptoms and workplace functioning would improve posttreatment. We further estimated the cost savings incurred by observed changes in work-related outcomes.

## Methods

### Study Setting and Participants

This naturalistic study analyzed repeated measures retrospective data from n=11,131 patients enrolled in SilverCloud within Ireland’s HSE during the time period in which the WPAI questionnaire was administered (from March 8, 2023 to May 27, 2024). Note that this study adhered to CHEERS (Consolidated Health Economic Evaluation Reporting Standards) 2022 guidelines for economic evaluations (see Checklist 1) [34]. Within the HSE, patients are referred to SilverCloud through health professionals across 5 referral groups (ie, General Practitioners, Primary Care Psychology, Counseling in Primary Care, Community Mental Health, and an HSE-funded mental health charity called Jigsaw). The HSE and SilverCloud staff both provided communications to these referral groups detailing the availability of this iCBT service, who would be suitable for it, and how to have a referral conversation with their patients.

 When considering a patient for SilverCloud, health professionals perform an initial screening to assess whether they may be suitable for the intervention. Eligible patients for the treatment (and the study) were at least 18 years old and presented with common mental health issues (eg, low mood, anxiety, feelings of isolation, stress, poor sleep). Patients were ineligible if they had severe mental health symptoms such as manic or psychotic episodes and/or suicidal ideation. If a patient is suitable, the health professional submits a referral to SilverCloud, which triggers an email invite sent to the patient through the SilverCloud platform. Within the email, an invitation link redirects the client to the sign-up page on the SilverCloud website. Patients who do not activate their account receive weekly reminder emails for the first 30 days following the initial invitation. During the sign-up process, patients are asked to provide demographic information, complete baseline clinical questionnaires, and can choose to consent for their pseudoanonymized data to be used for clinical research and service evaluation purposes. Once sign-up is complete, patients are directed to the SilverCloud homepage where they can begin the program.

### Intervention

Within the HSE, SilverCloud’s *Space from Depression*, *Space from Anxiety*, *Space from Depression and Anxiety*, and *Space from Generalized Anxiety Disorder* programs were offered during the study time period. These are general purpose iCBT programs that consist of a series of modules aligned with evidence-based CBT principles for the treatment of depression and anxiety [33,35]. Specifically, each program aims to help the patient develop and increase an understanding of the relationship between one’s thoughts, feelings, and behaviors, alongside information about coping strategies and therapeutic techniques such as graded exposure, behavioral activation, cognitive restructuring, problem solving, and activity scheduling. This content is delivered through various interactive media and tools, including quizzes, videos, activities, and personal stories. During program use, patients also complete routine outcome measures (ie, Patient Health Questionnaire-9 [PHQ-9], Generalized Anxiety Disorder-7 [GAD-7], WPAI, see below) at intake, week 2, week 4, and week 8 of treatment. Risk is monitored continuously through item 9 on the PHQ-9.

Patients are also assigned a supporter during their use of SilverCloud. All supporters are graduate psychologists with at least a master’s degree or doctorate in progress who have been trained in the delivery of online support. Supporters also receive training specific to providing support on SilverCloud and receive supervision from clinical supervisors. Supporters provide an asynchronous written “review” in the platform to the patient. The primary aim of the review is to provide the patient with personalized guidance and feedback based on their progress and clinical symptomology. Supporter reviews are provided weekly for the first 6 weeks, though additional reviews may be provided at the discretion of the supporter and their clinical supervisor. Once all the reviews are provided, or if the patient does not engage with the program for 3 consecutive review periods, the supporter discharges the patient from the supported service and no longer provides reviews. After patients are discharged, they still have full access to the platform in an unsupported capacity.

### Assessments

#### Demographic Questionnaire

During intake, clients completed demographic questions about gender, education, race, ethnicity, age, work status, and marital status. They are also asked whether they are in concurrent mental health treatment and to rate how likely they think SilverCloud will work for them.

#### Routine Outcome Measures

The following questionnaires are part of the HSE’s routine outcome measures that were administered at intake, week 2, week 4, and week 8 of treatment.

##### WPAI: Specific Health Problem

The WPAI: Specific Health Problem is a 6-item questionnaire that probes the impact of a specified health problem on workplace functioning over the past 7 days [19]. Outcomes include absenteeism (work time missed), presenteeism (impairment while working), overall productivity loss (absenteeism and presenteeism combined), and nonwork activity impairment (see Multimedia Appendix 1). Each outcome is expressed as a percentage, with higher numbers indicating greater impairment. The measure has shown good construct validity and test-retest reliability [19].

##### Patient Health Questionnaire-9

The PHQ-9 is a 9-item self-report questionnaire for depressive symptoms, with good reliability, sensitivity, and specificity [36]. The total score ranges from 0 to 27, with each item rated on a 4-point Likert scale (0=*not at all* to 3=*nearly every day*). The scale has 4 cut-off points of 5, 10, 15, and 20, which represent mild, moderate, moderately severe, and severe levels of depression, respectively.

##### Generalized Anxiety Disorder-7

The GAD-7 is a 7-item self-report questionnaire that identifies individuals with generalized anxiety disorder [37]. Items are scored on a 4-point Likert scale (0=*not at all* to 3=*nearly every day*). The total score can range from 0 to 21, with three cut-off points (5, 10, and 15) representing mild, moderate, and severe anxiety levels, respectively. The scale has also demonstrated good reliability, sensitivity, and specificity [37].

### Ethical Considerations

All procedures of this study adhered to the ethical standards of the relevant national and institutional committees on human experimentation and the Declaration of Helsinki (1975, revised in 2008). The protocol-related documents and informed consent were reviewed and approved by the Trinity College Dublin School of Psychology Research Ethics Committee (Approval ID: SPREC112021-04). Approval for users to not provide informed consent was granted, as consent had already been given for treatment and for therapists to gather and examine clinical outcome measures as part of normal service evaluation procedures. Service users who refused consent to having their data passed on for these purposes were not included in this study. Study data were collected and stored in a deidentified manner and complied with General Data Protection Regulations. Patients were not compensated for this study, as the data were derived from routine service assessments administered during routine treatment. No images of patients were used in this study or are presented in this manuscript or supplemental materials.

### Data Analysis

The study sample size was based on all available data from all patients who reported being employed via the WPAI questionnaire. Each patient’s last assessment prior to treatment discharge was used as their follow-up measure. We included all patients who completed the baseline assessment and used multiple imputation to estimate follow-up CMD and workplace functioning scores for those missing follow-up data. Performing the Little Missing Completely At Random test suggested WPAI data were not missing at random (*χ*^2^_27_=3921.24, *P*<.001). Multiple imputation is a conservative method for handling missing data as it incorporates natural variability into the missing values [38]. Pooled estimates from the imputation are reported. For sensitivity purposes, core analyses were repeated using complete-case data (Multimedia Appendix 2). Descriptive analyses were conducted on patients’ demographics, baseline clinical and work-related outcomes, as well as treatment characteristics.

First, univariate regressions were used to test whether CMD (depression and anxiety) (independent variable) and workplace functioning (dependent variable) were associated at baseline. To identify potential confounds, we tested the association between changes in WPAI outcomes and covariates, including demographics (age, gender, education, ethnicity, marital status, long-term conditions) and treatment-related characteristics (concurrent treatment, expectation), which were coded according to Table 1. Due to our naturalistic study design, the time from baseline to follow-up varied considerably (mean 34.89, SD 14.68 d). We controlled for this by evaluating the assessment interval as a potential covariate, which captured the treatment duration relevant to the measured effects. Next, to assess whether workplace functioning and CMD improved posttreatment, we employed mixed-effects models, regressing workplace functioning and CMD outcomes (dependent variable) on time as a fixed effect (independent variable), including significant covariates previously identified. To address repeated observations nested within patients across time, we incorporated random intercepts for patients in our models. To quantify the pre-post change in WPAI outcomes, we calculated Cohen *d* effect sizes [39]. We explored baseline CMD severity and comorbidity as moderators to examine their effects on changes in workplace functioning.

To calculate productivity-related cost savings from the intervention, we adopted the human capital approach, a widely established method for estimating the monetary value associated with productivity losses. The method values 1 hour of productivity loss based on the individual’s hourly wages [40]. We obtained median hourly salary estimates (stratified by gender and education) from available Irish census data in the 2022 Structure of Earnings Survey [41]. Thus, estimates are in 2022 € values (€1=approximately US $1.05) [42]. We then multiplied these estimates by the percentages of absenteeism, presenteeism, and overall productivity loss to determine the cost, scaling it accordingly to weekly and yearly estimates, assuming a full-time 39-hour work week. We did not apply a discount rate given the short timeframe of the study. We calculated this per patient and used the mean for analysis.

All analyses were conducted in R (version 4.4.0), and all tests were 2-sided, with statistical significance set at *α*=.05.

**Table 1.** Baseline characteristics of the study sample^a^.

Characteristics	Participants
Age (y), n (%)	
18‐24	1461 (20.64)
25‐64	5578 (78.82)
65+	38 (0.54)
Gender, n (%)	
Female	4956 (70.23)
Male	2067 (29.29)
Other/Prefer not to say	34 (0.48)
Race and ethnicity, n (%)	
White Irish	6023 (85.32)
Other White European	652 (9.24)
Asian	99 (1.40)
Mixed	74 (1.05)
Black	71 (1.01)
Latino	62 (0.88)
Other	78 (1.10)
Education, n (%)	
Primary to secondary	2411 (34.41)
College/university	3614 (51.58)
Postgraduate	982 (14.01)
Marital status, n (%)	
Single	2134 (30.30)
In a relationship	2332 (33.12)
Married	2155 (30.60)
Separated/divorced	376 (5.34)
Widowed	45 (0.64)
Long-term condition, n (%)	
Yes	1605 (22.87)
No	5413 (77.13)
Concurrent treatment, n (%)	
Yes	3766 (53.34)
No	3295 (46.66)
Depression severity (PHQ-9)^b^, mean (SD)	12.82 (6)
Minimal, n (%)	546 (7.66)
Mild, n (%)	1832 (25.71)
Moderate, n (%)	1982 (27.82)
Moderately severe, n (%)	1621 (22.75)
Severe, n (%)	1144 (16.06)
Anxiety severity (GAD-7)^c^, mean (SD)	12.62 (5.38)
Minimal, n (%)	457 (6.41)
Mild, n (%)	1830 (25.68)
Moderate, n (%)	1903 (26.71)
Severe, n (%)	2935 (41.19)
WPAI^d^ absenteeism (%), mean (SD)	19.45 (33.88)
WPAI presenteeism (%), mean (SD)	34.17 (30.04)
WPAI productivity loss (%), mean (SD)	49.19 (34.44)
WPAI activity impairment (%), mean (SD)	49.74 (27.87)
Treatment duration (d), mean (SD)	34.89 (14.68)
Treatment expectation (0‐4), mean (SD)	2.17 (0.65)

a Summary descriptives were calculated based on the total responses per variable, which may differ due to missing data. The proportion of missingness for all variables was less than 2.5%, except for baseline presenteeism (6%), productivity loss (6%), and activity impairment (8%).

b PHQ-9: Patient Health Questionnaire-9.

c GAD-7: Generalized Anxiety Disorder-7.

d WPAI: Workplace Productivity and Activity Impairment.

## Results

### Patient Characteristics

**Figure 1.**
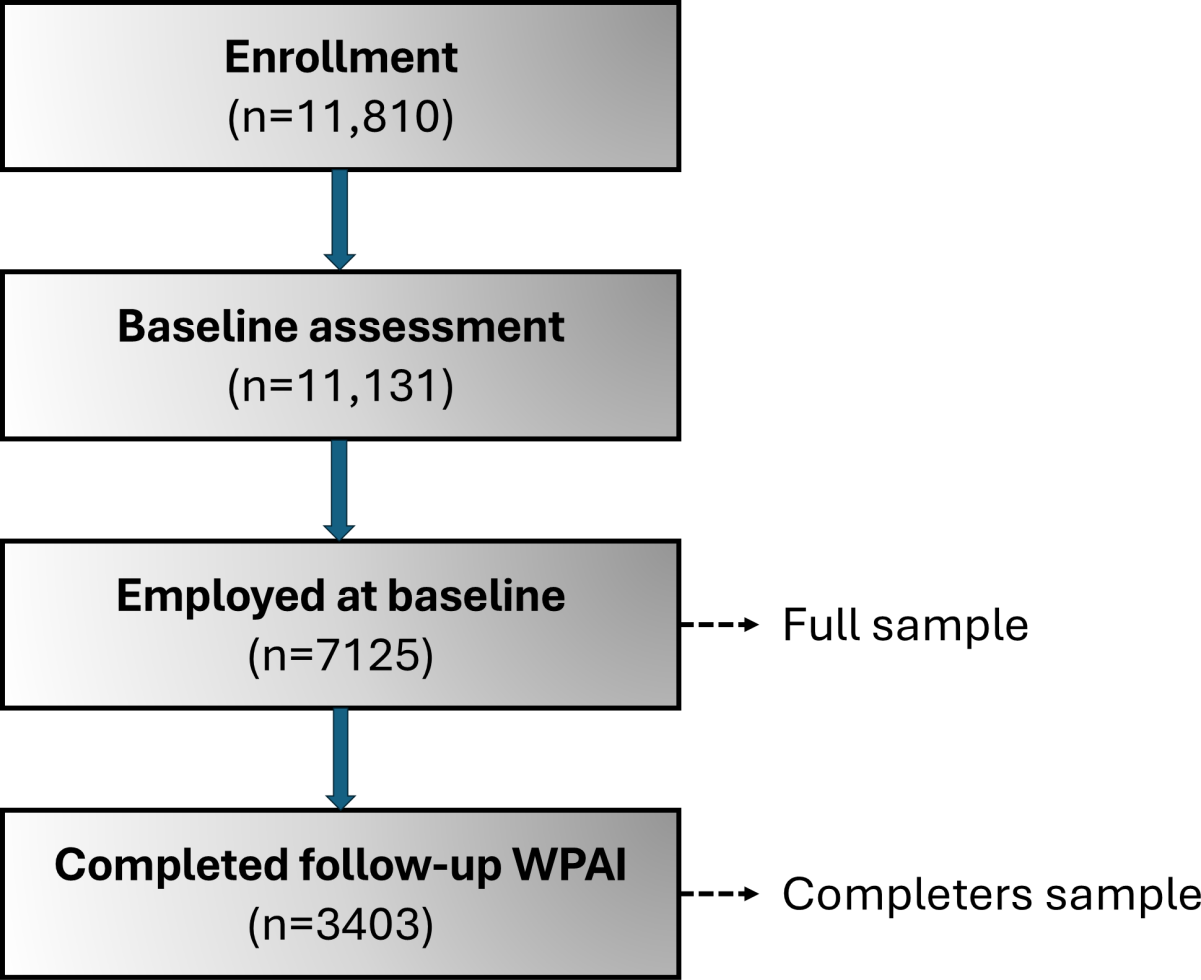
Participant flow chart. Participants may have completed their baseline and follow-up assessments fully or partially, as completion of the assessment was not compulsory for them to progress in the treatment. WPAI: Work Productivity and Activity Impairment.

The participant flow chart is shown in Figure 1. Of the 11,810 patients enrolled in the national iCBT service, 11,131 completed the baseline assessment fully or partially, of whom 7125 (64%) reported being employed. These 7125 participants formed the final sample for analyses.

### Baseline Analyses

Table 1 outlines the characteristics of our sample. Patients were primarily 25 to 64 years old, female, White Irish, had college or university education, in romantic relationships, and not presenting with a physical long-term condition. Their baseline severity on average fell within the moderate classification of depression (PHQ-9) and anxiety (GAD-7), respectively. Over half were receiving concurrent treatment in addition to iCBT, which most were undertaking for approximately 35 days. Overall, there was a positive expectation regarding the benefit that users believed they would gain from the treatment, with approximately 90% indicating that they believed that it was at least ’somewhat likely’ to work for them. Regarding workplace functioning, patients were on average absent from work 20% of the week and had 34% reduced performance while working during the week. Both productivity loss and activity impairment averaged near 50%.

A series of univariate regressions revealed positive associations between both PHQ-9 and GAD-7 and all four WPAI outcomes at baseline (Table 2), where greater levels of depression and anxiety predicted increased absenteeism, presenteeism, overall productivity loss, and activity impairment (all *P*<.001). More specifically, every 1-point increase on the PHQ-9 and GAD-7 predicted approximately a 1% to 2.6% decrease in workplace functioning.

**Table 2.** Baseline associations between clinical and work-related outcomes at baseline, as assessed using univariate linear regressions.

Work-related outcomes and CMD^a^	Univariate analyses			
	Unstandardized β (95% CI)	SE	*t* test (*df*)	*P* value
Absenteeism				
PHQ-9^b^	1.36 (1.24‐1.48)	0.06	20.89 (7119.64)	<.001
GAD-7^c^	1.01 (0.87‐1.15)	0.07	13.76 (7120.21)	<.001
Presenteeism				
PHQ-9	1.43 (1.31‐1.54)	0.06	24.28 (2451.73)	<.001
GAD-7	1.56 (1.42‐1.70)	0.07	23.34 (1358.33)	<.001
Productivity loss				
PHQ-9	2.30 (2.18‐2.42)	0.06	36.38 (3893.65)	<.001
GAD-7	2.13 (1.99‐2.27)	0.07	28.84 (1972.90)	<.001
Activity impairment				
PHQ-9	2.55 (2.45‐2.65)	0.05	52.26 (1090.67)	<.001
GAD-7	2.26 (2.14‐2.38)	0.06	39.88 (3198.07)	<.001

a CMD: common mental disorder.

b PHQ-9: Patient Health Questionnaire-9.

c GAD-7: Generalized Anxiety Disorder-7.

### Pre-Post Changes in Workplace Functioning

**Figure 2.**
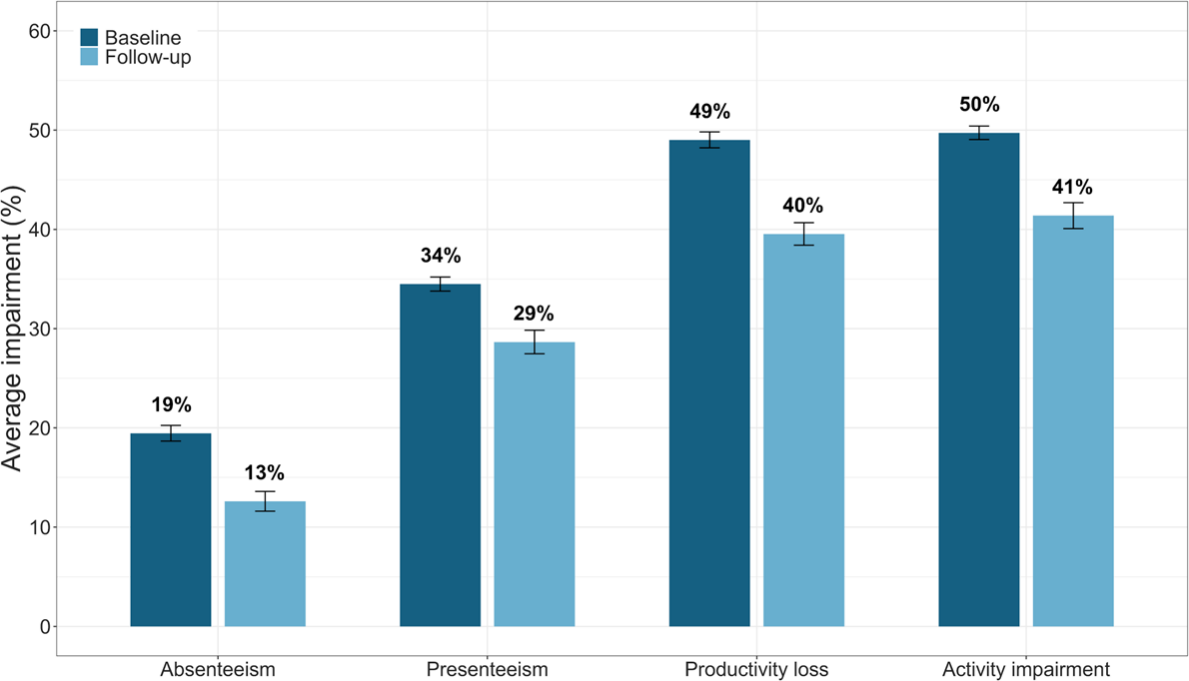
Pooled mean impairment at baseline and follow-up for absenteeism, presenteeism, productivity loss, and activity impairment. Error bars denote the 95% CI.

Over an average treatment period of 35 days, we observed significant improvements across all WPAI outcomes. Specifically, there was an average reduction of 6.85% in absenteeism (*b*=−6.85, *t*_71.47_=−12.75, *P*<.001, 95% CI −5.79 to −7.91, Cohen *d*=0.21), 5.84% in presenteeism (*b*=−5.84, *t*_38.51_=−9.15, *P*<.001, 95% CI −4.59 to −7.09, Cohen *d*=0.18), 9.48% in productivity loss (*b*=−9.48, *t*_61.18_=−15.70, *P*<.001, 95% CI −8.30 to −10.66, Cohen *d*=0.27), and 8.34% in activity impairment (*b*=−8.34, *t*_29.13_=−12.88, *P*<.001, 95% CI −7.07 to −9.61, Cohen *d*=0.30) (Figure 2). Covariates such as treatment duration, treatment expectation, gender, concurrent treatment, and marital status were significant predictors of change in one or more WPAI outcomes. Importantly, all observed pre-post changes in WPAI outcomes remained statistically significant after adjusting for these confounders (all *P*<.001) (Multimedia Appendix 3).

For depression and anxiety, we further observed a significant reduction in both PHQ-9 (*b*=−2.70, *t*_42.71_=-27.11, *P*<.001, 95% CI −2.50 to −2.90, Cohen *d*=0.51) and GAD-7 (*b*=−2.71, *t*_39.36_=−26.52, *P*<.001, 95% CI −2.51 to −2.91, Cohen *d*=0.52) scores posttreatment. Partial correlations accounting for significant covariates previously identified revealed that these clinical symptom reductions were also positively associated with changes in absenteeism (PHQ-9: *r*=0.10, 95% CI 0.07 to 0.13; GAD-7: *r*=0.06, 95% CI 0.03 to 0.09), presenteeism (PHQ-9: *r*=0.18, 95% CI 0.14 to 0.21; GAD-7: *r*=0.21, 95% CI 0.18 to 0.24), productivity loss (PHQ-9: *r*=0.21, 95% CI 0.18 to 0.24; GAD-7: *r*=0.21, 95% CI 0.18 to 0.24), and activity impairment (PHQ-9: *r*=0.34, 95% CI 0.31 to 0.37; GAD-7: *r*=0.33, 95% CI 0.30 to 0.36; all *P*<.001). This pattern indicates that greater improvements in depression and anxiety symptoms were associated with greater improvements in each WPAI outcome.

**Figure 3.**
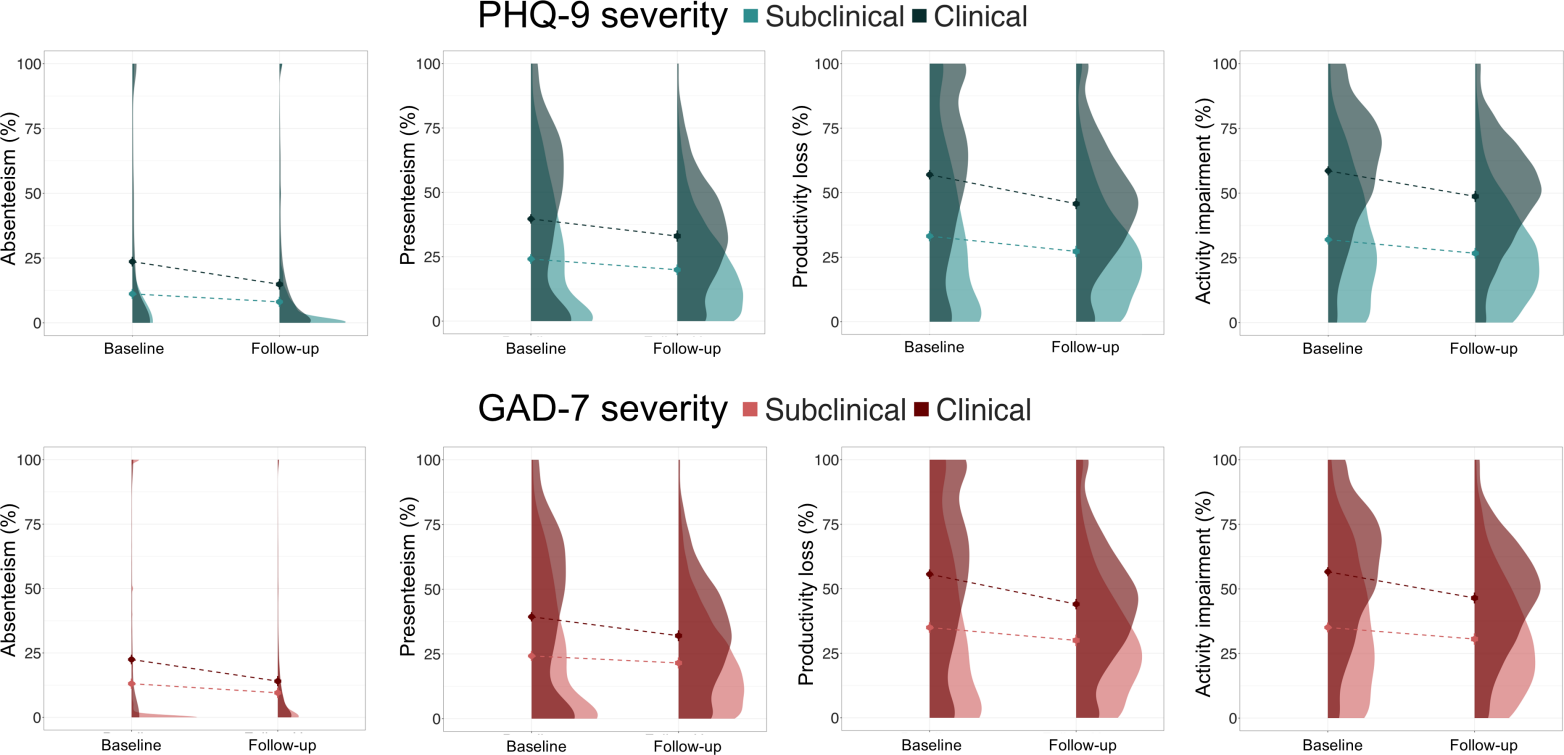
Changes in work-related outcomes from baseline to follow-up, moderated by baseline depression severity (top row) and baseline anxiety severity (bottom row). The points represent unadjusted means, and the error bars represent SE. GAD7: Generalized Anxiety Disorder-7; PHQ9: Patient Health Questionnaire-9*.*

To examine whether baseline CMD severity, that is, clinical (moderate to severe symptoms) vs subclinical (minimal to mild symptoms), influences treatment effects on workplace functioning, we included baseline PHQ-9 and GAD-7 scores as moderators, along with covariates previously identified for each outcome (see Multimedia Appendix 4 for demographic characteristics by CMD severity). Significant time × baseline PHQ-9 interaction effects suggested that patients with higher baseline depression experienced larger reductions in absenteeism (*b*=−5.68, *t*_130.37_=-5.55, 95% CI −7.68 to −3.68; *P*<.001), presenteeism (*b*=−2.48, *t*_50.02_=−2.03, 95% CI −4.87 to −0.09; *P*=.048), productivity loss (*b*=−5.32, *t*_80.23_=−4.43, 95% CI −6.35 to −2.87; *P*<.001), and activity impairment (*b*=−4.61, *t*_107.97_=−5.17, 95% CI −9.61 to −7.07; *P*<.001). More specifically, those who were clinical at baseline experienced larger reductions than their subclinical counterparts in absenteeism (subclinical: *b*=−3.06, 95% CI −1.59 to −4.53, *P*<.001; clinical: *b*=−8.74, 95% CI −7.35 to −10.13, *P*<.001), presenteeism (subclinical: *b*=−4.18, 95% CI −2.55 to −5.81, *P*<.001; clinical: *b*=−6.67, 95% CI −4.95 to −8.39, *P*<.001), productivity (subclinical: *b*=−5.93, 95% CI −4.13 to −7.73, *P*<.001; clinical: *b*=−11.25, 95% CI −9.72 to −12.78, *P*<.001), and activity impairment (subclinical: *b*=−5.27, 95% CI −3.80 to −6.74, *P*<.001; clinical: *b*=−9.88, 95% CI −8.35 to −11.41, *P*<.001; Figure 3 and Multimedia Appendix 4).

A similar trend was also observed for anxiety, where those entering treatment with higher GAD-7 scores experienced larger reductions in workplace functioning (absenteeism: *b*=−4.87, *t*_380.35_=−5.21, 95% CI −6.69 to −3.05, *P*<.001; presenteeism: *b*=−4.62, *t*_76.03_=−4.17, 95% CI −6.80 to −2.44, *P*<.001; productivity loss: *b*=−6.61, *t*_102.34_=−5.70, 95% CI −8.88 to −4.34, *P*<.001; activity impairment: *b*=−5.67, *t*_75.85_=−5.90, 95% CI −7.55 to −3.79, *P*<.001). Patients with clinical score levels on the GAD-7 had larger decreases than subclinical patients in absenteeism (subclinical: *b*=−3.54, 95% CI −1.99 to −5.09, *P*<.001; clinical: *b*=−8.41, 95% CI −7.16 to −9.66, *P*<.001), presenteeism (subclinical: *b*=−2.70, 95% CI −0.96 to −4.44, *P*=.02; clinical: *b*=−6.19, 95% CI −4.64 to −7.74, *P*<.001), productivity loss (subclinical: *b*=−4.99, 95% CI −3.09 to −6.89, *P*<.001; clinical: *b*=−11.60, 95% CI −10.19 to −13.01, *P*<.001), as well as activity impairment (subclinical: *b*=−4.50, 95% CI −2.79 to −6.21, *P*<.001; clinical: *b*=−10.20, 95% CI −8.73 to −11.67, *P*<.001) (Figure 3 and Multimedia Appendix 4).

Importantly, we found that patients experiencing comorbid clinical levels of depression and anxiety at baseline also improved more in workplace functioning than those who were noncomorbid. This was evidenced by significant time × comorbidity interaction effects observed for all WPAI outcomes (all *P*<.001, Multimedia Appendix 5).

### Cost Analysis

Based on 2022 Irish census median salary estimates by gender and educational attainment, the average median salary in the sample was estimated to be €836.06 (95% CI €830.29-€841.83, SD €248.62) per week, which is, €43,475.34 (95% CI €43,175.15-€43,775.43, SD €12,928.16) per year. At baseline, we calculated the cost induced by deficits in workplace functioning for each patient (WPAI % multiplied by salary), before totaling these values to produce an average. The average costs of absenteeism and presenteeism per patient per week were estimated to be €159.82 (95% CI €153.00-€166.64, SD €293.59) and €290.80 (95% CI €284.30-€297.30, SD €280.04), totaling to an annual monetary loss of €8310.64 (95% CI €7955.52-€8664.48, SD €15,266.39) and €15,121.60 (95% CI €14,782.87-€15,459.13, SD €14,562.22). For overall productivity loss, the total cost induced is nearly half of each patient’s earnings, estimated at €409.87 (95% CI €402.41-€417.33, SD €321.29) per week and €21,313.26 (95% CI €20,925.32-€21,701.20, SD €16,707.25) per year.

**Figure 4.**
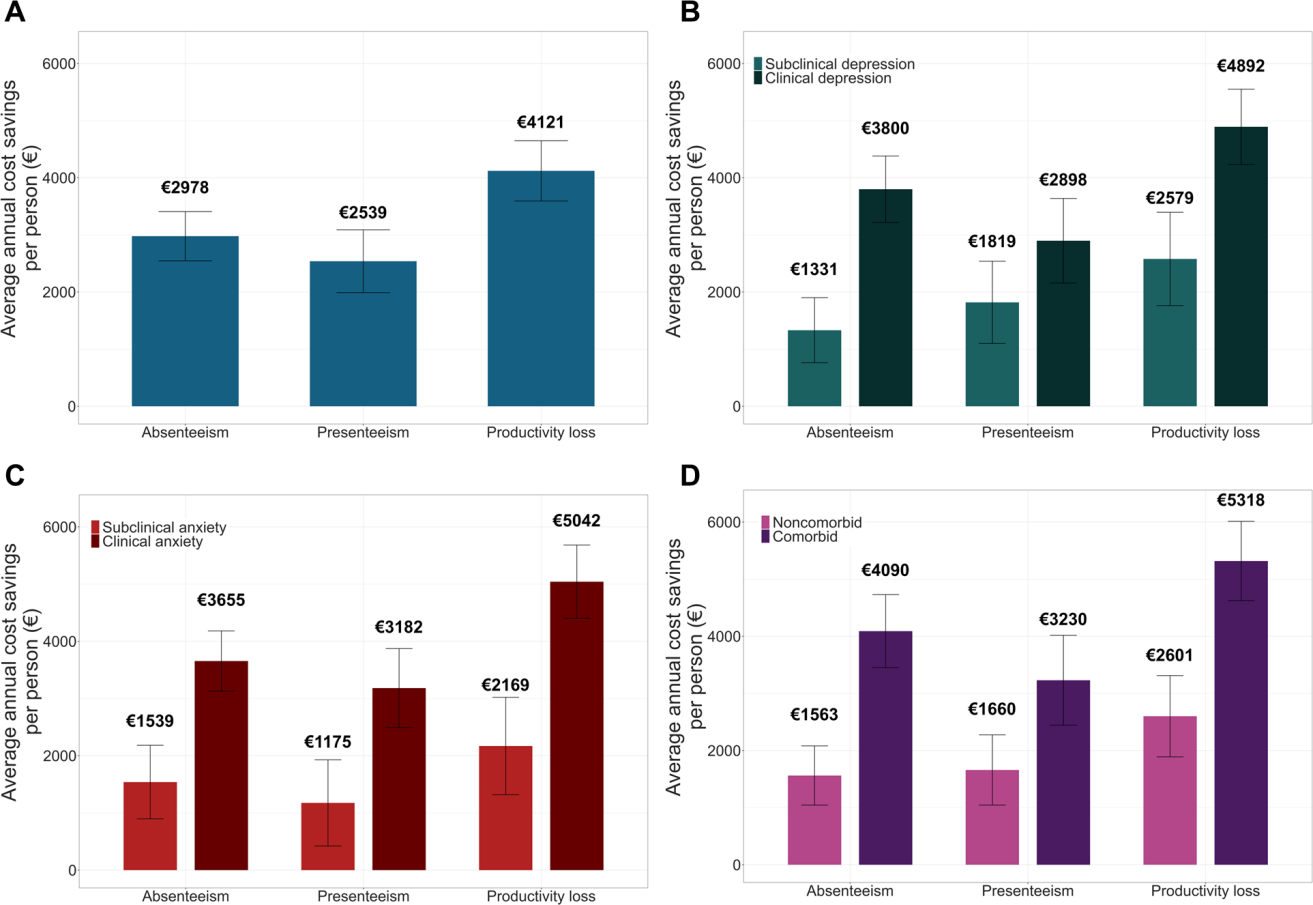
(A) Estimated average annual cost savings (in 2022 € values; €1=approximately US $1.05) postintervention induced by improvements in absenteeism, presenteeism, and overall productivity loss for patients with clinical levels versus subclinical levels of (B) depression, (C) anxiety, and for (D) patients with comorbid clinical levels of depression and anxiety vs those without. Monetary figures were rounded to the nearest integer. Error bars denote the 95% CI.

To estimate the annual cost savings due to improvements in workplace functioning, we multiplied the adjusted mean reduction in absenteeism (6.85%), presenteeism (5.84%), and productivity loss (9.48%) by the average salary of our sample. With each percent change in WPAI outcomes, annual costs were estimated to decline by €434.75 per patient. This results in annual cost savings of €2978.06 for absenteeism (95% CI €2546.94-€3409.18), €2538.96 for presenteeism (95% CI €1989.67-€3088.25), and €4121.11 for overall productivity loss (95% CI €3589.11-€4645.11) for each patient treated (Figure 4A). In the context of our study sample (n=7125), this improvement equates to an annual cost saving of €29.33 million.

Importantly, when we consider CMD severity and comorbidity of patients at baseline, cost savings were more substantial for those presenting with more severe symptomatology (Figure 4). Considering overall productivity loss, annual cost savings for patients with clinical depression (11.25% improvement) were 1.90 times higher compared to subclinical patients (5.93% improvement) (average cost savings for clinical depression: €4892.98, 95% CI €4233.28-€5550.72 vs subclinical depression: €2579.04, 95% CI €1762.23-€3395.77; Figure 4B). For anxiety, annual cost savings for clinical patients (11.60% improvement) were 2.33 times higher than subclinical patients (4.99% improvement) for overall productivity loss (average cost savings for clinical anxiety: €5042.36, 95% CI €4402.67-€5682.71 vs subclinical anxiety: €2169.02, 95% CI €1317.28-€3020.76; Figure 4C). Similarly, cost savings are 2.05 times higher for comorbid patients (12.23% improvement) compared to their counterparts (5.98% improvement) (average cost savings for non-comorbid: €2601.92, 95% CI €1890.44-€3313.40 vs comorbid: €5318.41, 95% CI €4623.67-€6013.15; Figure 4D).

### Sensitivity Analysis

To determine whether multiple imputation introduced bias into our findings, we repeated the core analysis, including only patients who completed both baseline and follow-up assessments (n=3402) (Multimedia Appendix 2). The results remained consistent, showing significant improvements in all CMD and WPAI outcomes, with greater improvements among patients with clinical levels of CMD and comorbid CMD compared to their counterparts.

## Discussion

This study is the first to use nationwide routine care data to investigate the effectiveness of iCBT, delivered as a public health initiative for treating CMD, in improving workplace functioning in the general population. These improvements in work productivity were estimated to lead to substantial cost savings. As hypothesized, patients reported fewer missed workdays, less impairment during work and activities, and higher overall productivity after 5 weeks of treatment. Although the effects observed here (6%‐10% reductions) were smaller than those in prior studies that included small, completer-only samples with limited generalizability [22,29], they align more closely with those reported in meta-analyses of work-focused CBT or iCBT interventions on work absences and effectiveness (Hedges *g*=0.22‐0.25) [25,43]. Work-related improvements were observed alongside reductions in depression and anxiety symptoms, which were consistent with our previous study [32].

Notably, iCBT improved workplace functioning for both clinical and subclinical patients, with greater benefits for those with more severe symptomatology at baseline. This finding not only highlights iCBT as an effective intervention for improving workplace functioning in individuals with clinically apparent symptoms but also for subclinical individuals, helping to prevent their symptoms from reaching clinical levels. Note that these subclinical individuals likely represent the typical working population [22]. Future research should explore whether engagement with standard vs work-focused content differentially impacts improvements in workplace functioning, again using more representative data from the general working population. This is particularly pertinent given the limited evidence supporting the superiority of tailored over nontailored digital interventions [26,27,44].

The consequences and scale of impaired workplace functioning due to mental illness present major public health challenges, necessitating the implementation of cost-effective and accessible interventions [3,44]. Our study builds on the promising evidence of iCBT interventions in improving clinical, work-related, and financial outcomes in occupational contexts [22-24] and demonstrates the scalability of these benefits when iCBT is provided in a national health service. By expanding the intervention’s reach beyond industry-specific settings and barriers, these outcomes have significant implications for the economic health of the nation. This is evident in the measurable cost savings demonstrated in our study, estimated at more than €4000 over the course of a year per patient treated, totaling €29 million annually within our sample. These findings are aligned with the conclusions of the few economic evaluations on workplace iCBT interventions [22-24,29]. The findings also dovetail with other mental health and economic evaluations focusing on iCBT implemented within national health care systems, such as MindSpot in Australia and SilverCloud within Talking Therapies in the United Kingdom, though these prior studies were not focused on cost-effectiveness through workplace productivity [33,45]

These cost-savings estimates should also be seen as conservative, given that they focus on costs due to workplace impairment and do not capture medical costs, costs of providing the treatment, or broader societal benefits (eg, reduced welfare and health care expenditure, better health care utilization, and positive spillover effects on patients’ support networks) [3,46]. While it is possible that expanding treatment coverage in the population may increase the average cost per person initially, particularly after reaching previously inaccessible patients in remote areas, the expected financial returns from improved health and work-related outcomes have been shown to far outweigh the treatment costs for depression and anxiety (return ratio of 2.3-3:1 considering only economic benefits, 3.3‐5.7:1 when including health returns) [3]. Furthermore, our findings highlight that iCBT is uniquely positioned to bridge the substantial treatment gap between demand and availability on a national level. As a feasible, scalable solution, iCBT can alleviate the strain of CMDs on overburdened health care systems and improve workforce productivity, all the while allowing greater access to care at lower cost compared to in-person approaches [47,48].

This study has several limitations. The naturalistic design of our study, without comparison groups, prevents us from determining the causality of the observed treatment effects. Estimates from this study could serve as benchmarks for future studies focused on evaluating outcomes and the cost-effectiveness of mental health treatments within the HSE. Outcomes were also collected at fixed intervals set by the service, with the latest assessment administered at week 8 of treatment. Thus, the extent to which treatment effects on symptoms persist over longer time frames (eg, 6 mo, 1 y) is unknown in this sample. This may particularly bias study estimates projecting annual cost savings. Though prior work investigating the treatment durability of the SilverCloud iCBT program suggests that approximately 70% of patients who recover from symptoms remain in recovery 9 months later [49]. The study also relies on self-report data, uncorroborated by objective and formal assessments, which may be more accurate. Another limitation is the precision of our estimates due to the nature of the available data. As WPAI outcomes can only be calculated for employed individuals [19], this fails to account for those unemployed due to their condition (ie, complete loss of productivity).

Additionally, while using national salary estimates is a common procedure for estimating cost savings [22-24,29], collecting and incorporating data on patient-specific salary, employment sector, and typical hours worked would produce financial return estimates produced directly from actual participant wage and job data. It is also important to note that we assumed a linear transformation of productivity loss to monetary loss, and it is possible that the association may be nonlinear. Data on intervention costs and other medical costs were also unavailable, which prevented a more comprehensive cost-effectiveness analysis. Future studies should incorporate income and medical cost assessments with comparator treatments using representative data to enable a national economic evaluation.

Depression and anxiety significantly impact labor participation and productivity, but this can be effectively managed with accessible treatments through public health services. Building on work that primarily focused on work productivity improvements from iCBT in occupational contexts, this study provides evidence that iCBT for the general adult population in a national routine health care setting is associated with improvements in both CMD and workplace productivity losses associated with CMD. These productivity improvements were further associated with sizable cost savings. These findings suggest that integrating iCBT into national mental health infrastructure can be a feasible, scalable solution, which could generate clinical and economic benefits at a population level.

## Supplementary material

10.2196/80689 Multimedia Appendix 1 Calculation of work productivity activity and impairment outcomes.

10.2196/80689 Multimedia Appendix 2 Core data analyses for completers sample.

10.2196/80689 Multimedia Appendix 3 Covariate analyses examining pre-post changes in Work Productivity and Activity Impairment (WPAI).

10.2196/80689 Multimedia Appendix 4 Pre-post analyses of changes in Work Productivity and Activity Impairment (WPAI) for patients with clinical and subclinical common mental disorders.

10.2196/80689 Multimedia Appendix 5 Pre-post analyses of changes in Work Productivity and Activity Impairment (WPAI) for patients with comorbid and noncomorbid common mental disorders.

10.2196/80689 Checklist 1 CHEERS 2022 checklist.

## Abbreviations

CBT: cognitive behavioral therapy
CHEERS: Consolidated Health Economic Evaluation Reporting Standards
CMD: common mental disorder
GAD-7: Generalized Anxiety Disorder-7
HSE: Health Service Executive
iCBT: internet-based cognitive behavioral therapy
PHQ-9: Patient Health Questionnaire-9
WPAI: Work Productivity and Activity Impairment
