# Supplementary material for: Changes in Workplace Productivity and Estimated Cost Savings During Internet-Based Cognitive Behavioral Therapy in the Irish National Health Service: Naturalistic, Repeated-Measures, Retrospective Survey Study
Source: J Med Internet Res. 2026 Apr 7;28:e80689. doi: 10.2196/80689 (PMC13054933; doi:10.2196/80689)
Supplement: Multimedia Appendix 1 [file jmir-v28-e80689-s001.docx]

# Multimedia Appendix 1. Calculation of Work Productivity Activity and Impairment Outcomes

Work Productivity Activity and Impairment ((Reilly et al., 1993) outcomes are expressed as percentages, where higher values indicate more impairment and lower productivity. The items are as follows:

1. Are you currently employed?
2. How many hours did you miss due to the specified problem?
3. How many hours did you miss for other reasons?
4. How many hours did you actually work?
5. How much did the problem affect your productivity while working?
6. How much did the problem affect your regular activities?

***Absenteeism*** = Percentage of work time missed due to the problem:

- Q2 / (Q2 + Q4)

***Presenteeism*** = Percentage of impairment while working due to the problem:

- Q5 / 10

***Overall Productivity Loss*** = Percentage of work impairment due to the problem:

- Q2 / (Q2 + Q4) + [(1 - (Q2 / (Q2 + Q4))) x (Q5 / 10)]

***Activity Impairment*** = Percentage of activity impairment due to the problem:

- Q6 / 10

Scores are converted to percentages by multiplying by 100.
