# Supplementary material for: Changes in Workplace Productivity and Estimated Cost Savings During Internet-Based Cognitive Behavioral Therapy in the Irish National Health Service: Naturalistic, Repeated-Measures, Retrospective Survey Study
Source: J Med Internet Res. 2026 Apr 7;28:e80689. doi: 10.2196/80689 (PMC13054933; doi:10.2196/80689)
Supplement: Multimedia Appendix 2 [file jmir-v28-e80689-s002.docx]

# Multimedia Appendix 2. Core data analyses for completers sample

As a sensitivity check, we repeated our core analyses including data from patients who completed both their baseline and follow-up assessment fully or partially (N=3402) (see **Table 1** for patient characteristics of the completers sample).

## Table 1. Baseline characteristics of the completers sample.

| **Characteristics** | **N** | **%** |
| --- | --- | --- |
| Age (year) |  |  |
| 18-24 | 583 | 17.27 |
| 25-64 | 2777 | 82.28 |
| 65+ | 15 | 0.44 |
| Gender |  |  |
| Female | 2373 | 70.50 |
| Male | 975 | 28.97 |
| Other/Prefer Not to Say | 18 | 0.53 |
| Race & Ethnicity |  |  |
| White Irish | 2867 | 85.15 |
| Other White European | 339 | 10.07 |
| Mixed | 34 | 1.01 |
| Latino | 33 | 0.98 |
| Black | 33 | 0.98 |
| Asian | 31 | 0.92 |
| Other | 30 | 0.89 |
| Education |  |  |
| Primary to Secondary | 1069 | 31.99 |
| College/University | 1750 | 52.36 |
| Postgraduate | 523 | 15.65 |
| Marital Status |  |  |
| Single | 932 | 27.75 |
| In a Relationship | 1068 | 31.80 |
| Married | 1163 | 34.62 |
| Separate/Divorced | 172 | 5.12 |
| Widowed | 24 | 0.7 |
| Long-Term Condition |  |  |
| Yes | 864 | 25.82 |
| No | 2482 | 74.18 |
| Concurrent Treatment |  |  |
| Yes | 1818 | 54.07 |
| No | 1544 | 45.93 |
| **Characteristics** | **Mean** | **(SD)** |
| Depression Severity (PHQ-9) | 12.53 | 5.94 |
| Minimal, N, % | 283 | 8.32 |
| Mild, N % | 896 | 26.34 |
| Moderate, N, % | 959 | 28.19 |
| Moderately Severe, N, % | 775 | 22.78 |
| Severe, N, % | 489 | 14.37 |
| Anxiety Severity (GAD-7) | 12.4 | 5.3 |
| Minimal, N, % | 227 | 6.67 |
| Mild, N % | 883 | 25.96 |
| Moderate, N, % | 938 | 27.57 |
| Severe, N, % | 1354 | 39.80 |
| WPAI Absenteeism (%) | 18.82 | 33.60 |
| WPAI Presenteeism (%) | 33.53 | 29.72 |
| WPAI Productivity Loss (%) | 48.36 | 34.39 |
| WPAI Activity Impairment (%) | 49.26 | 27.60 |
| Treatment Duration (days) | 34.89 | 14.68 |
| Treatment Expectation (0-4) | 2.24 | 0.64 |

Note: Summary descriptives were calculated as per the total response per variable, which may differ with regards to missing data. Missingness proportion for all variables were <2.7%, except for baseline presenteeism (6%), productivity loss (6%), and activity impairment (7%). PHQ-9: Patient Health Questionnaire-9; GAD-7: Generalized Anxiety Disorder-7; WPAI: Workplace Productivity and Activity Impairment.

**Baseline Analyses:** At baseline, similar to our main analyses, we found positive associations between the CMD scores (PHQ-9 and GAD-7) and WPAI scores, where higher depression and anxiety were associated with increased absenteeism [PHQ9: b=1.28, SE=0.09, p<.001; GAD7: b=0.95, SE=0.11, p<.001)], presenteeism [PHQ9: b=1.45, SE=0.09, p<.001; GAD7: b=1.58, SE=0.09, p<.001)], productivity loss [PHQ9: b=2.32, SE=0.09, p<.001; GAD7: b=2.13, SE=0.11, p<.001)], and activity impairment [PHQ9: b=2.61, SE=0.07, p<.001; GAD7: b=2.26, SE=0.08, p<.001)].

**Pre-post Analyses:** We observed significant improvements across all four WPAI outcomes, consistent to our main analyses. Findings showed an average reduction of 6.08% (SD=29.95) in absenteeism (b=-6.70, SE=0.53, p<.001), 4.88% (SD=31.10) in presenteeism (b=-4.60, SE=0.57, p<.001), 8.94% (SD=33.0) in productivity loss (b=-9.38, SE=0.61, p<.001), and 9.26% (SD=27.46) in activity impairment (b=-9.21, SE=0.49, p<.001), all with small effect sizes (Cohens’ *d* = .16-.34). After controlling for significant covariates including treatment duration, treatment expectation, and marital status, all observed improvements remained statistically significant (all p<.001). We further observed significant reductions in depression (PHQ9: M=3.01, SD=5.20, b=-3.01, SE=0.09, p<.001) and anxiety (GAD7: M=3.09, SD=5.17, b=-3.09, SE=0.09, p<.001). Similar to our main analyses, these reductions were positively associated with improvements in absenteeism [PHQ-9 r(3125)=0.12; GAD-7 r(3079)=0.08], presenteeism [PHQ-9 r(2771)=0.20; GAD-7 r(2727)=0.23], productivity loss [PHQ-9 r(2771)=0.25; GAD-7 r(2727)=0.27], and activity impairment [PHQ-9 r(3013)=0.35; GAD-7 r(2969)=0.35], with all p<.001.

Including baseline clinical severity (PHQ9 and GAD7) as a moderator in our analyses, findings revealed similar patterns of results where patients higher baseline depression experienced larger reductions in absenteeism (b=-4.43, SE=1.12, p<.001), productivity loss (b=-4.11, SE=1.29, p=.001), and activity impairment (b=-5.95, SE=1.03, p<.001), but not presenteeism (b=-1.61, SE=1.21, p=0.18). Clinical patients in depression experienced larger reductions than subclinical patients in absenteeism (subclinical: b=3.81, SE=.90, p=.001; clinical: b=8.24, SE=.67, p<.001), productivity (subclinical: b=6.69, SE=1.04, p<.001; clinical: b=10.79, SE=.76, p<.001), and activity impairment (subclinical: b=5.29, SE=.84, p<.001; clinical: b=11.23, SE=.60, p<.001). This was also the case for anxiety [absenteeism (b=-3.46, SE=1.14, p=.002), presenteeism (b=-4.66, SE=1.23, p<.001), productivity loss (b=-5.87, SE=1.31, p<.001), activity impairment (b=-6.28, SE=1.04]. p<.001) Patients with clinical level of GAD-7 at baseline improved more than subclinical patients in absenteeism (subclinical: b=4.38, SE=.94, p<.001; clinical: b=7.84, SE=.65, p<.001), presenteeism (subclinical: b=1.53, SE=1.01, p=.43; clinical: b=6.19, SE=.70, p<.001), productivity loss (subclinical: b=5.53, SE=1.07, p<.001; clinical: b=11.40, SE=.75, p<.001), as well as activity impairment (subclinical: b=5.04, SE=.86, p<.001; clinical: b=11.33, SE=.60, p<.001). A similar trend was also found for comorbidity, evidenced by significant time x comorbidity interaction effects observed for absenteeism (b=-4.56, SE=1.07, p<.001; subclinical: b=4.21, SE=0.79, p<.001; clinical: b=8.77, SE=0.86, p=.006), presenteeism (b=-3.31, SE=1.15, p=.004; subclinical: b=2.79, SE=0.79, p<.001; clinical: b=6.10, SE=0.78, p<.001;), productivity (b=-5.68, SE=1.23, p<.001; subclinical: b=6.31, SE=0.91, p<.001; clinical: b=12.00, SE=0.83, p<.001), and activity impairment (b=-5.86, SE=0.98, p<.001; subclinical: b=6.04, SE=0.73, p<.001; clinical: b=11.90, SE=0.66, p<.001).

**Cost-Saving Analyses**: Based on the gender and educational attainment of our completers sample, the average median salary is approximately €851.72 (SD=254.25) per week, i.e. €44,289.60 (SD=13,221.21) per year. Indirect costs induced by baseline absenteeism and presenteeism per patient per week are approximately €157.69 (SD=297.44) and €288.76 (SD=280.58), i.e., €8200, (SD=15,466.95) and 15,015 (SD=€14,590) respectively per year. Combined, the total indirect cost induced by productivity loss is nearly half of each patient’s earnings (€413.27 per week (SD=€329.10) and €21,490 (SD=€17,112.96) per year). To estimate cost-savings as a result of the intervention, we multiplied the adjusted mean reduction in absenteeism (6.71%), presenteeism (4.67%), and productivity loss (9.45%) from the previous analysis by the estimated average salary of our sample. Each % change in WPAI outcomes was associated with a cost reduction of €442.90. Therefore annual cost-savings were estimated at €2970.00 for absenteeism, €2066.79 for presenteeism, and €4183.88 for overall productivity loss for each patient treated. In the context of our completers sample (N=3402), this improvement is equal to an annual cost-saving of approximately €14.2 million. Similar to our main analyses, cost-savings were significantly greater for those who present with more severe symptomatology. Specifically concerning productivity loss, patients with clinical depression compared to subclinical patients had 2.16 times higher cost-savings resulting from improved overall productivity (monetary difference: €1821.87). For anxiety, cost savings for clinical patients were 2.06 times higher (monetary difference: €2599.85) compared to subclinical patients. Those presenting with comorbid CMD also had higher cost-savings than non-comorbid patients (1.77 times higher, €2299.43 difference).
