# Supplementary material for: Changes in Workplace Productivity and Estimated Cost Savings During Internet-Based Cognitive Behavioral Therapy in the Irish National Health Service: Naturalistic, Repeated-Measures, Retrospective Survey Study
Source: J Med Internet Res. 2026 Apr 7;28:e80689. doi: 10.2196/80689 (PMC13054933; doi:10.2196/80689)
Supplement: Multimedia Appendix 3 [file jmir-v28-e80689-s003.docx]

# Multimedia Appendix 3. Covariate analyses examining pre-post changes in WPAI

## Table 2. Pre-post changes in workplace productivity outcomes controlling for significant covariates.

| **Workplace Outcomes** | **Predictors** | **Beta** | **Standard Error** | **T** | **p** |
| --- | --- | --- | --- | --- | --- |
| *Absenteeism* | | | | | |
|  | Treatment | -6.85 | 0.54 | -12.75 | <.001 |
|  | Gender |  |  |  |  |
|  | Male | 1.93 | 0.80 | 2.42 | 0.16 |
|  | Other | 9.85 | 5.37 | 1.84 | 0.07 |
|  | Civil Status |  |  |  |  |
|  | Married | -0.93 | 0.91 | -1.02 | 0.31 |
|  | Separated/Divorce | 6.93 | 1.77 | 3.91 | <.001 |
|  | Single | -0.26 | 0.90 | -0.29 | 0.77 |
|  | Widowed | -1.13 | 4.24 | -0.27 | 0.79 |
| *Presenteeism* | | | | | |
|  | Treatment | -5.84 | 0.64 | -9.15 | <.001 |
|  | Married | -7.73 | 0.81 | -9.58 | <.001 |
|  | Separated/Divorce | -6.03 | 1.45 | -4.14 | <.001 |
|  | Single | 1.22 | 0.83 | 1.47 | 0.14 |
|  | Widowed | -10.14 | 4.03 | -2.52 | 0.01 |
| *Productivity Loss* | | | | | |
|  | Treatment | -9.48 | 0.60 | -15.70 | <.001 |
|  | Treatment Duration | -0.05 | 0.03 | -1.66 | 0.10 |
| *Activity Impairment* | | | | | |
|  | Treatment | -8.34 | 0.65 | -12.88 | <.001 |
|  | Concurrent Treatment (Yes) | 7.52 | 0.65 | 11.63 | <.001 |
|  | Treatment Duration | -0.02 | 0.03 | -0.70 | 0.48 |
|  | Treatment Expectation | -4.51 | 0.53 | -8.49 | <.001 |

##

## Table 3. Estimated marginal means of workplace outcomes at baseline and follow-up controlling for significant covariates.

| **Workplace Outcomes** | **Baseline Mean (SE)** | **Follow-Up Mean (SE)** |
| --- | --- | --- |
| *Absenteeism* | 23.69 (2.02) | 16.84 (2.02) |
| *Presenteeism* | *32.34 (0.85)* | *26.50 (0.98)* |
| *Productivity Loss* | *49.01 (0.40)* | *39.54 (0.58)* |
| *Activity Impairment* | *49.48 (0.34)* | *41.14 (0.67)* |
|  |  |  |
