# Supplementary material for: Changes in Workplace Productivity and Estimated Cost Savings During Internet-Based Cognitive Behavioral Therapy in the Irish National Health Service: Naturalistic, Repeated-Measures, Retrospective Survey Study
Source: J Med Internet Res. 2026 Apr 7;28:e80689. doi: 10.2196/80689 (PMC13054933; doi:10.2196/80689)
Supplement: Multimedia Appendix 4 [file jmir-v28-e80689-s004.docx]

# Multimedia Appendix 4. Pre-post analyses of changes in WPAI for patients with clinical and subclinical CMD

## Table 4. Demographic characteristics by CDM.

| **Characteristics** | PHQ-9 | | GAD-7 | | Comorbid PHQ-9 & GAD-7 | |
| --- | --- | --- | --- | --- | --- | --- |
|  | Subclinical | Clinical | Subclinical | Clinical | No | Yes |
|  | **N (%)** | **N (%)** | **N (%)** | **N (%)** | **N (%)** | **N (%)** |
| **Age (year)** |  |  |  |  |  |  |
| 18-24 | 321 (13.61%) | 1,140 (24.16%) | 290 (12.76%) | 1,171 (24.37%) | 456 (24.62%) | 1,005 (25.39%) |
| 25-64 | 2,010 (85.21%) | 3,568 (75.63%) | 1,953 (85.96%) | 3,625 (75.44%) | 2,631 (84.35%) | 2,947 (74.46%) |
| 65+ | 28 (1.19%) | 10 (0.21%) | 29 (1.28%) | 9 (0.19%) | 32 (1.03%) | 6 (0.15%) |
| **Gender** |  |  |  |  |  |  |
| Female | 1,621 (68.8%) | 3,335 (70.94%) | 1,546 (68.23%) | 3,410 (71.18%) | 2,136 (68.59%) | 2,820 (71.52%) |
| Male | 729 (30.94%) | 1,338 (28.46%) | 7,13 (31.47%) | 1,354 (28.26%) | 969 (31.12%) | 1,098 (27.85%) |
| Other/Prefer Not to Say | 6 (0.25%) | 28 (0.60%) | 7 (0.31%) | 27 (0.56%) | 9 (0.29%) | 25 (0.63%) |
| **Race & Ethnicity** |  |  |  |  |  |  |
| White Irish | 2,011 (85.43%) | 4,012 (85.27%) | 1,935 (85.43%) | 4,088 (85.27%) | 2,655 (85.34%) | 3,368 (85.31%) |
| Other White European | 223 (9.47%) | 429 (9.12%) | 205 (9.05%) | 447 (9.32%) | 306 (9.84%) | 346 (8.76%) |
| Asian | 37 (1.57%) | 62 (1.32%) | 34 (1.50%) | 65 (1.36%) | 44 (1.41%) | 55 (1.39%) |
| Mixed | 17 (0.72%) | 57 (1.21%) | 21 (0.93%) | 53 (1.11%) | 23 (0.74%) | 51 (1.29%) |
| Black | 20 (0.85%) | 51 (1.08%) | 29 (1.28%) | 42 (0.88%) | 31 (1.00%) | 40 (1.01%) |
| Latino | 21 (0.89%) | 41 (0.87%) | 20 (0.88%) | 42 (0.88%) | 24 (0.77%) | 38 (0.96%) |
| Other | 25 (1.06%) | 53 (1.13%) | 21 (0.93%) | 57 (1.09%) | 28 (0.90%) | 50 (1.26%) |
| **Education** |  |  |  |  |  |  |
| Primary to Secondary | 663 (28.43%) | 1,748 (37.39%) | 695 (30.92%) | 1,716 (36.06%) | 938 (30.36%) | 1,473 (37.61%) |
| College/University | 1,238 (53.09%) | 2,376 (50.82%) | 1,165 (51.82%) | 2,449 (51.46%) | 1,625 (52.59%) | 1,989 (50.78%) |
| Postgraduate | 431 (18.48%) | 551 (11.79%) | 388 (17.26%) | 594 (12.48%) | 527 (17.06%) | 455 (11.62%) |
| **Marital Status** |  |  |  |  |  |  |
| Single | 574 (24.42%) | 1,560 (33.26%) | 632 (27.99%) | 1,502 (31.40%) | 846 (27.26%) | 1,288 (32.70%) |
| In a Relationship | 691 (29.39%) | 1,641 (34.98%) | 567 (25.11%) | 1,765 (36.89%) | 877 (28.26%) | 1,455 (36.94%) |
| Married | 942 (40.07%) | 1,213 (25.86%) | 892 (39.50%) | 1,263 (26.40%) | 1,185 (38.19%) | 970 (24.63%) |
| Separate/Divorced | 122 (5.19%) | 254 (5.41%) | 141 (6.24%) | 235 (4.91%) | 167 (5.38%) | 209 (5.31%) |
| Widowed | 22 (0.94%) | 23 (0.49%) | 26 (1.15%) | 19 (0.4%) | 28 (0.90%) | 17 (0.43%) |
| **Long-Term Condition** |  |  |  |  |  |  |
| Yes | 461 (19.72%) | 1,144 (24.44%) | 523 (23.29%) | 1,082 (22.67%) | 667 (21.61%) | 938 (23.86%) |
| No | 1,877 (80.28%) | 3,536 (75.56%) | 1,723 (76.71%) | 3,690 (77.33%) | 2,419 (78.39%) | 2,994 (76.14%) |
| **Concurrent Treatment** |  |  |  |  |  |  |
| Yes | 1,015 (43.21%) | 2,751 (58.38%) | 1,068 (47.17%) | 2,698 (56.24%) | 1,447 (46.56%) | 2,319 (58.66%) |
| No | 1,334 (56.79%) | 1,961 (41.62%) | 1,196 (52.83%) | 2,099 (43.76%) | 1,661 (53.44%) | 1,634 (41.34%) |

## Table 5. Estimated marginal means of workplace outcomes in patients with subclinical and clinical levels of depression and anxiety at baseline and follow-up.

|  | **Depression (PHQ-9)** | | | **Anxiety (GAD-7)** | | |
| --- | --- | --- | --- | --- | --- | --- |
| **Workplace Outcomes** | **Clinical Status** | **Baseline Mean (SE)** | **Follow-Up Mean (SE)** | **Clinical Status** | **Baseline Mean (SE)** | **Follow-Up Mean (SE)** |
| *Absenteeism* | | | | | | |
|  | Subclinical | 15.20 (2.07) | 12.14 (2.07) | Subclinical | 17.45 (2.07) | 13.91 (2.09) |
|  | Clinical | 27.77 (2.01) | 19.03 (2.05) | Clinical | 27.02 (2.02) | 18.61 (2.04) |
| *Presenteeism* | | | | | | |
|  | Subclinical | 22.98 (0.96) | 18.79 (1.03) | Subclinical | 23.41 (0.95) | 20.71 (1.14) |
|  | Clinical | 37.62 (0.87) | 30.95 (1.14) | Clinical | 37.63 (0.89) | 30.31 (1.04) |
| *Productivity Loss* | | | | | | |
|  | Subclinical | 33.16 (0.66) | 27.23 (0.87) | Subclinical | 34.97 (0.69) | 29.98 (0.89) |
|  | Clinical | 56.95 (0.48) | 45.70 (0.73) | Clinical | 55.65 (0.48) | 44.05 (0.67) |
| *Activity Impairment* | | | | | | |
|  | Subclinical | 32.55 (0.55) | 27.28 (0.73) | Subclinical | 35.49 (0.56) | 30.99 (0.85) |
|  | Clinical | 58.12 (0.39) | 48.23 (0.79) | Clinical | 56.16 (0.40) | 46.00 (0.75) |
