# Supplementary material for: Changes in Workplace Productivity and Estimated Cost Savings During Internet-Based Cognitive Behavioral Therapy in the Irish National Health Service: Naturalistic, Repeated-Measures, Retrospective Survey Study
Source: J Med Internet Res. 2026 Apr 7;28:e80689. doi: 10.2196/80689 (PMC13054933; doi:10.2196/80689)
Supplement: Multimedia Appendix 5 [file jmir-v28-e80689-s005.docx]

# Multimedia Appendix 5. Pre-post analyses of changes in WPAI for patients with comorbid and non-comorbid CMD

## Table 6. Linear mixed effect model estimates of pre-post changes in workplace productivity outcomes for patients with comorbid clinical levels of depression and anxiety vs. non-comorbid patients, controlling for significant covariates.

| **Workplace Outcomes** | **Predictors**  **Post-Hoc Paired Contrast** | **Beta** | **Standard Error** | **T/T-ratio** | **p** |
| --- | --- | --- | --- | --- | --- |
| *Absenteeism* | | | | | |
|  | Stage*Comorbid_Yes | -5.81 | 0.97 | -6.01 | <.001 |
|  | Comorbid_No:Baseline-Follow-Up | 3.60 | 0.66 | 5.46 | <.001 |
|  | Comorbid_Yes:Baseline-Follow-Up | 9.41 | 0.76 | 12.31 | <.001 |
| *Presenteeism* | | | | | |
|  | Stage*Comorbid_Yes | -3.61 | 1.08 | -3.33 | .001 |
|  | Comorbid_No:Baseline-Follow-Up | 3.82 | 0.71 | 5.35 | <.001 |
|  | Comorbid_Yes:Baseline-Follow-Up | 18.18 | 0.80 | 22.80 | <.001 |
| *Productivity Loss* | | | | | |
|  | Stage*Comorbid_Yes | -6.25 | 1.07 | -5.82 | <.001 |
|  | Comorbid_No:Baseline-Follow-Up | 5.98 | 0.82 | 7.32 | <.001 |
|  | Comorbid_Yes:Baseline-Follow-Up | 12.23 | 0.80 | 15.39 | <.001 |
| *Activity Impairment* | | | | | |
|  | Stage*Comorbid_Yes | -4.83 | 0.89 | -5.43 | <.001 |
|  | Comorbid_No:Baseline-Follow-Up | 5.64 | 0.74 | 7.61 | <.001 |
|  | Comorbid_Yes:Baseline-Follow-Up | 10.47 | 0.82 | 12.84 | <.001 |

##

## Table 7. Estimated marginal means of workplace outcomes in patients with comorbid clinical levels of depression and anxiety vs. non-comorbid patients with at baseline and follow-up.

| **Workplace Outcomes** | **Comorbidity** | **Baseline Mean (SE)** | **Follow-Up Mean (SE)** |
| --- | --- | --- | --- |
| *Absenteeism* | | | |
|  | Yes | 28.94 (2.02) | 19.53 (2.07) |
|  | No | 16.88 (2.04) | 13.28 (2.03) |
| *Presenteeism* | | | |
|  | Yes | 39.14 (0.89) | 31.71 (1.15) |
|  | No | 24.78 (0.91) | 20.96 (1.00) |
| *Productivity Loss* | | | |
|  | Yes | 59.04 (0.52) | 46.81 (0.75) |
|  | No | 36.30 (0.58) | 30.32 (0.76) |
| *Activity Impairment* | | | |
|  | Yes | 59.73 (0.43) | 49.25 (0.82) |
|  | No | 36.67 (0.48) | 31.02 (0.72) |
